# “Chasing Rainbows” Beyond Kaposi Sarcoma’s Dermoscopy: A Mini-Review

**DOI:** 10.3390/dermatopathology11040035

**Published:** 2024-11-25

**Authors:** Emmanouil Karampinis, Olga Toli, Georgia Pappa, Anna Vardiampasi, Melpomeni Theofili, Efterpi Zafiriou, Mattheos Bobos, Aimilios Lallas, Elizabeth Lazaridou, Biswanath Behera, Zoe Apalla

**Affiliations:** 1 Second Dermatology Department, School of Health Sciences, Aristotle University of Thessaloniki, 54124 Thessaloniki, Greece; bethlaz@auth.gr (E.L.); zoimd@yahoo.gr (Z.A.); 2 Department of Dermatology, Faculty of Medicine, School of Health Sciences, University General Hospital of Larissa, University of Thessaly, 41110 Larissa, Greece; zafevi@o365.uth.gr; 3 Department of Dermatology, Oncoderm Center One Day Clinic, 45332 Ioannina, Greece; olgatolimail@gmail.com; 4 2nd Department of Dermatology and Venereology, “Attikon” General University Hospital, Medical School, National and Kapodistrian University of Athens, 12462 Athens, Greece; gpappa100@gmail.com (G.P.); melpomenitheofili@hotmail.com (M.T.); 5 Agioi Anargyroi General and Oncologic Hospital of Kifisia, 14564 Athens, Greece; annavardiampasi@yahoo.gr; 6 Microdiagnostics Pathology Laboratory, 54622 Thessaloniki, Greece; mbobos@icloud.com; 7 First Dermatology Department, School of Health Sciences, Aristotle University of Thessaloniki, 54124 Thessaloniki, Greece; emlallas@gmail.com; 8 Department of Dermatology and Venereology, All India Institute of Medical Sciences (AIIMS), Bhubaneswar 751019, Odisha, India; biswanathbehera61@gmail.com

**Keywords:** dermoscopy, dermatopathology, skin cancer, rainbow pattern, polychromatic pattern

## Abstract

The dermoscopic rainbow pattern (RP), also known as polychromatic pattern, is characterized by a multicolored appearance, resulting from the dispersion of polarized light as it penetrates various tissue components. Its separation into different wavelengths occurs according to the physics principles of scattering, absorption, and interference of light, creating the optical effect of RP. Even though the RP is regarded as a highly specific dermoscopic indicator of Kaposi’s sarcoma, in the medical literature, it has also been documented as an atypical dermoscopic finding of other non-Kaposi skin entities. We aim to present two distinct cases—a pigmented basal cell carcinoma (pBCC) and an aneurysmatic dermatofibroma—that exhibited RP in dermoscopy and to conduct a thorough review of skin conditions that display RP, revealing any predisposing factors that could increase the likelihood of its occurrence in certain lesions. We identified 33 case reports and large-scale studies with diverse entities characterized by the presence of RP, including skin cancers (Merkel cell carcinoma, BCC, melanoma, etc.), adnexal tumors, special types of nevi (blue, deep penetrating), vascular lesions (acroangiodermatitis, strawberry angioma, angiokeratoma, aneurismatic dermatofibromas, etc.), granulation tissue, hypertrophic scars and fibrous lesions, skin infections (sporotrichosis and cutaneous leishmaniasis), and inflammatory dermatoses (lichen simplex and stasis dermatitis). According to our results, the majority of the lesions exhibiting the RP were located on the extremities. Identified precipitating factors included the nodular shape, lesion composition and vascularization, skin pigmentation, and lesions’ depth and thickness. These parameters lead to increased scattering and interference of light, producing a spectrum of colors that resemble a rainbow.

## 1. Introduction

A rainbow pattern (RP) or polychromatic pattern is described as a multicolored area in dermoscopy, believed to result from the separation of the polarized light, through its route into the different tissue components, into different wavelengths [1]. RP is characterized by a more structured and harmonious display of colors with a specific visual arrangement. It is considered to be a highly specific dermoscopic finding of Kaposi’s sarcoma mainly in the nodular or papular subtype, due to the angled diffraction and interference of the polarized light to the vascular structural “back-to-back” arrangement of the multifocal tumor [2,3]. Another theory supporting the RP observed in Kaposi sarcoma is the presence of numerous hyaline globule aggregates [4].

Beyond Kaposi sarcoma, the range of lesions showing the RP on dermoscopy has expanded to include various other conditions. From skin cancer such as basal cell carcinoma (BCC) and melanoma to inflammatory diseases such as stasis dermatitis and lichen planus to scars and various vascular tumors, skin lesions with different consistencies may display this dermoscopic sign [1,5]. However, the frequency of its appearance is not characteristic of the respective lesion but a rare finding.

Therefore, we want to make a comprehensive review, listing all the skin lesions that were reported in the medical literature, and exhibit RP as well as investigate any predisposing factors that may increase the likelihood of this pattern appearing in lesions.

## 2. Case Presentations

### 2.1. Case 1

**Figure 1:**
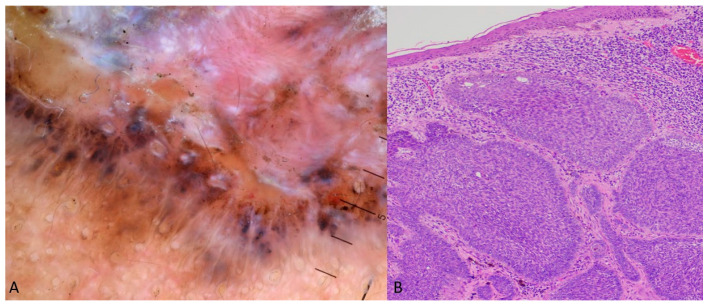
Dermoscopic (**A**) and histopathology (**B**) (H&E, 4Χ magnification) images of a nodular pBCC, which displayed PR during the dermoscopy examination.

A dark-skinned Indian patient visited our department concerned about a slowly growing pigmented lesion on their face that had been present for one year. The dermoscopic image revealed a pink-brownish stroma, and pigmented structures in the periphery can be observed. RP can be recognized in the upper part of the image (Figure 1). The histopathology of the lesion revealed nests of basaloid cells with cytological atypia, apoptotic bodies, mitotic figures, and characteristic peripheral palisading confirming the histopathology of BCC (Figure 1). There is a dermal chronic inflammatory cell infiltrate with pigment incontinence also present.

### 2.2. Case 2

**Figure 2:**
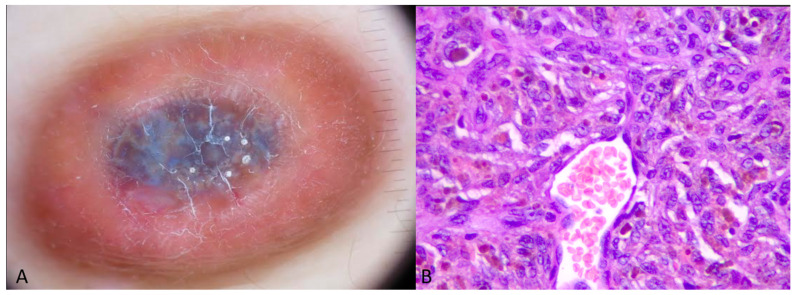
Dermoscopy of a hemosiderotic dermatofibroma, displaying RP in the center of the lesion: (**A**) histopathology of the tumor showed dense fibrohistiocytic proliferation, scattered hemosiderin deposition, and a vessel filled with erythrocytes (**B**) (H&E 20X magnification).

A young Caucasian girl presented with a single, asymptomatic, raised, and pigmented nodule. The dermoscopic image revealed a fine peripheral pigment network and a central bluish-purple homogeneous area with areas of RP (Figure 2). Histopathology revealed spindle cells with a vaguely fascicular architecture along with associated scattered multinucleated giant cells with peripheral cytoplasmic deposits of hemosiderin. A mild chronic inflammatory infiltrate, of predominantly lymphocytes and histiocytes, is also identified. Cytological atypia, necrosis, or mitotic figures are not identified (Figure 2).

## 3. Methods

We conducted a literature review from inception until August 2024. We searched for cases of skin lesions with RP under the dermoscope, examining the characteristics of these lesions and the authors’ hypotheses regarding this phenomenon. It is worth mentioning the distinction between the terms “multicolor” and “rainbow pattern”. While both terms involve the presence of multiple colors, the “rainbow pattern” is characterized by a unique iridescence, whereas “multicolor” refers, for example, to the various levels of pigmentation. Taking this differentiation into account, we focused exclusively on the rainbow pattern, due to its distinctive feature. Factors such as the patient’s phototype, location, and nature of the lesion, as well as other dermoscopic findings, were investigated to identify factors that may precipitate the appearance of RP in a lesion. The cases were identified through a thorough search of the PubMed database using the terms: [Rainbow pattern] AND [Dermoscopy] while only English-language studies were included.

## 4. Results

The search yielded a total of 33 results. From these studies and their citations, we identified 29 studies that discussed RP as a dermoscopic characteristic of lesions. Table 1 depicts the different lesion subtypes.

More precisely, we identified 4 patients with pseudo-Kaposi, also called acroangiodermatitis, in dark-skinned patients from Spain and Ecuador, all located at the legs [5,6]. Additional dermoscopic findings included polymorphic vessels, vascular lacunas, hemorrhagic crusts, white shiny structures, and rosettes. The authors suggested that the birefringence phenomenon was responsible for the appearance of shiny structures, including white shiny lines, white shiny areas, and rosettes. These features were attributed to the fibroblastic proliferation and are likely due to the interaction of polarized light with collagen [8]. The same phenomenon was proposed to explain the RP in these lesions, accounting for the frequent simultaneous appearance of both dermoscopic findings under polarized light. Therefore, it is proposed that the increased fibroblastic activity in pseudo-Kaposi sarcoma presenting as white shiny structures might be suggestive of pseudo-Kaposi diagnosis rather than Kaposi sarcoma in the case of shiny violaceous papules or plaques appearing with RP under dermoscopy. According to the images available, the RP was presented mainly in the center of the pseudo-Kaposi lesion [5,6].

Concerning vascular lesions, we also identified 5 patients presenting with pyogenic granuloma (3 located on the legs and 2 on the neck) with dermoscopy revealing red structureless areas, white intersecting lines, and a prominent RP predominantly in the center of the lesion. The majority of the cases from this category involved Turkish patients [5]. Other vascular lesions noted were strawberry angioma (1 patient), angiokeratoma (3 patients), aneurismatic dermatofibroma (2 patients), vascular tumors (such as intravascular papillary endothelial hyperplasia or Masson tumor (1 patient)), and a case of pleiomorphic vascular sarcoma (1 patient). All lesions described were raised or nodular, mostly appearing on legs or forearms [1,5,6,7,8,9].

Another lesion type exhibiting the rainbow pattern on dermoscopy was hypertrophic scars [1,2,20]. All case reports concerned leg scars with additional reports of post-infectious scars, specifically post-wart [20] and healing scar lesions following sporotrichosis and cutaneous leishmaniasis infections [23]. The authors attributed the RP in leg scars on dermoscopy to the unique vasculature of the limbs, combined with the scar vascularization and raised surface created by the hypertrophy. This configuration provides a suitable angle at which polarized light is diffracted and interferes within the vessels and other lesional structures [5]. Other tissues exhibiting RP were dermatofibromas (central RP) (2 patients) and granulοmatous lesions, such as granuloma annular and penile sclerosing granuloma (2 patients) [1,22]. Cutaneous inflammatory lesions also appeared in the list of conditions associated with RP, such as stasis dermatitis [2,6], lichen planus [6,22], skin infections (sporotrichosis and cutaneous leishmaniasis) [23], and dissecting cellulitis of the scalp [5]. It is worth mentioning that most of the inflammatory lesions, as well as the cases of penile sclerosing granuloma and acroangiodermatitis, were reported in dark-skinned patients (based on the macroscopic images presented in those cases). The higher melanin content in dark-toned patients can influence how light interacts with the skin; melanin can enhance the contrast of dermoscopic features, making the RP more noticeable against the darker background in case of inflammation.

**Figure 3:**
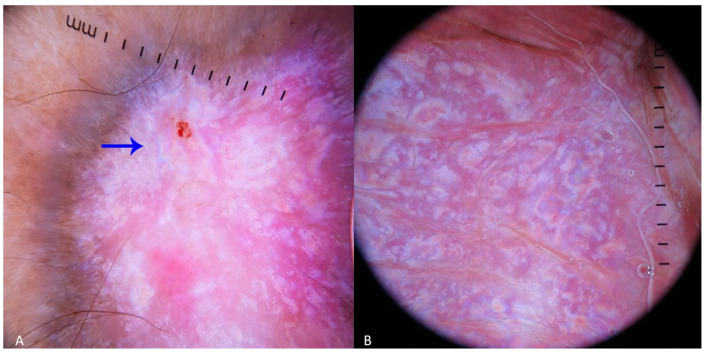
(**A**) Dermoscopy of Lichen planus in an Indian patient exhibiting PR (blue arrow) and peripheral pigmentation. (**B**) Dermoscopy of a post-burn hypertrophic scar presenting multiple iridescent areas.

Skin cancers (BCCs, melanoma, and Merkel cell carcinoma) are also represented in the RP list. While the RP may appear in dermoscopic images of skin cancer, it is not considered a typical feature. In the case of Merkel carcinoma, we identified two cases) that exhibited RP, all located on the extremities. The RP appeared peripherally in each case, with one instance where the pattern was surrounded by a reddish background. Polymorphous vessels and milky-red areas were also observed in dermoscopic images of Merkel cell carcinoma [13,14]. The RP observed in these lesions is thought to result from the chaotic neoangiogenesis occurring within the lesion. In cases of BCC, the RP was observed in both superficial and infiltrative subtypes, with the nodular subtype being the most common. Data suggest that certain BCC locations may predispose to the appearance of RP, while dermoscopic features such as white shiny areas often coexist, likely due to the similar effects of polarized light [9] (Figure 3). In a large study involving patients with skin phototypes IV-VI, the RP was observed in one-third of the cases, indicating that the dark phototype does play a role in PR appearance [8]. Data regarding BCC body sites are scattered, while most authors indicate the richness of vascularization between the islands of basaloid cells as the main reason for polarized light interactions. Based on available images, RP in BCCs appeared at the periphery or as peripheral shiny lobules within the lesion [1,8,9].

The RP in melanoma dermoscopy, aside from two case reports [1,6], has been studied mainly by two retrospective descriptive studies [10,11]. Both studies report that the presence of RP in melanoma is linked to tumor thickness. Focal RP was primarily associated with invasive melanoma, particularly those with a Breslow thickness ≥ 0.8 mm. Other dermoscopic features, including red-pink, blue-gray, and white coloration, blue-white veil, shiny white streaks, irregular vessels, blue-black pigmentation, and ulceration, were also correlated with thicker melanomas [10,11]. The depth of the elements that interact with the polarized light seems to have a contributing role in the presence of RP; apart from thick melanomas, it was also reported in deep penetrating nevi (1 case) [18] and blue nevus (3 cases) [1,17]. The deep penetrating nevus was reported in a Fitzpatrick IV patient with a nodular lesion, while blue nevus cases were presented as nodules on the legs and feet. Dermoscopy revealed blue to gray structureless areas, with the rainbow pattern appearing centrally in the lesion.

## 5. Discussion

The presence of the RP as a dermoscopic feature can be subjective, with varying opinions among authors regarding its presence in the same dermoscopic images [6,24]. From the cases and studies detected, we observed that some lesions were more likely to exhibit RP:

(a) Nodular lesions: This was also noted in Kaposi sarcomas, where papular and nodular subtypes presented RP, while bullous subtypes did not. In addition, nodular BCCs were more frequently associated with RP on dermoscopy compared to other subtypes, as well as nodular vascular lesions and hypertrophic scars.

(b) Lesions located on the extremities: The abundant vascular density of the lesions that arise on the legs may explain their higher likelihood of displaying RP. The second most frequent sites were hands and forearms, making extremity lesions more likely to exhibit that pattern on dermoscopy.

(c) Main components of the lesions—histopathology: RP was mainly observed in lesions with high vascular density or organized vascular lakes. It was also observed in skin cancers such as BCCs and melanomas with the tumor’s heterogeneous composition, localized vascular proliferation, variations in collagen and fibrosis, and uneven melanin distribution contributing to different interactions of polarized light. It is worth mentioning that, to the best of our knowledge, RP has not been observed in actinic keratosis or sebaceous hyperplasia, probably due to their low vascular density.

(d) Skin phototypes: Examples of inflammatory dermatoses, nevi, BCCs, and scars have been reported in dark-skinned individuals, with RP observed in some cases. In the case of BCC in the skin of color, race-specific dermoscopic features may also be present [25,26]. Figure 3 shows dermoscopic images of lichen planus and hypertrophic scar appearing in dark-skinned patients, verifying that dark skin is indeed a predisposing factor for a lesion to exhibit PR.

(e) Thickness of the lesion: RP is one of the dermoscopic features associated with melanoma thickness.

(f) Depth of the lesion: RP has been noted in deeper lesions, such as nodular BCCs, blue and penetrating nevi, and melanomas.

The parameters a, c, d, e, and f may be explained by the physics principles of polarized light and the basics of diffraction and interference [27] (Table 2).

The cases presented in the Case Presentations section also verify some of the predisposing factors mentioned above. The example with the nodular pBCC confirms that the shape of the lesion (nodular), histopathology (BCC structure), and skin color (dark-skinned) favor the appearance of RP as a component of the dermoscopic image (Figure 1). Similarly, the case of hemosiderotic dermatofibroma demonstrates that the lesion’s shape (nodular), histopathology (vascular subtype), and location (extremities) can also predispose to the appearance of RP in dermoscopy (Figure 2).

The physics hypothesis explaining the RP observed in dermoscopy may account for the findings in Table 2. For instance, in nodular lesions, the raised surface alters the angle of light incidence and exit, leading to variations in how light is bent and spread, contributing to the rainbow appearance. Concerning vascular laminas, the encounter of the light with these closely packed laminas causes multiple scattering events. This multiple scattering can enhance the PR by distributing light in various directions and wavelengths. A similar phenomenon can be observed in hematomas, where the blood contains varying shades and concentrations of hemoglobin that change over time, creating multiple scattering surfaces [27]. Finally, the higher melanin content in dark skin provides better contrast between the lesion and the surrounding skin [26]. However, those hypotheses must be confirmed with further studies and case observations.

In addition to the characteristics of the lesion, the technique used also plays a significant role. The authors in most cases also report that the application of a fluid, such as immersion oil or isopropyl alcohol, can enhance the effects of polarized light. We noticed that using isopropyl alcohol makes the RP more visible. However, RP has not been observed in any case using non-polarized dermoscopy, regardless of whether a fluid was applied [5].

Further research involving larger populations is necessary to validate these findings, as they currently arise from isolated cases and studies in the medical literature. Additionally, more studies are needed to investigate the implications of focal or peripheral RP, their histopathological correlations among various lesions, and their significance in identifying the malignant or vascular nature of a lesion.

## 6. Conclusions

RP can be detected in many skin lesions and should not only be considered a highly diagnostic finding for Kaposi sarcoma. Lesion characteristics such as form, depth and thickness, histopathology, and patient’s skin type may explain, with physics principles of diffraction and interference of the polarized light, the PR observed in their dermoscopic images.

## Figures and Tables

**Table 1:** Non-Kaposi skin lesions exhibiting RB on dermoscopy.

Non-Kaposi Lesions Exhibiting RP
Vascular lesions Strawberry angioma [1] Angiokeratoma [1,2] Aneurysmatic dermatofibroma [3,4] Pyogenic granuloma [1] Masson tumor [4] Pseudo-Kaposi [5,6] Cancerous lesions BCC [1,7,8,9] Melanoma [1,2,6,10,11] Pseudolymphoma [12] Merkel cell carcinoma [13,14] Atypical fibroxanthoma [15] Subungual metastasis [16] Nevi Blue nevi [1,17] Deep penetrating nevi [18] Adnexal tumors Sebaceous adenoma [19] Granulation and scaring lesions Hypertrophic scars [1,2,20] Granuloma annular [1] Penile sclerosing granuloma [21] Dermatofibromas [1] Inflammatory dermatoses Stasis dermatitis [2,6] Lichen planus [6,22] Dissecting cellulitis [1] Skin infections Cutaneous leishmaniasis [23] Sporotrichosis [23]

**Table 2:** Examples of non-Kaposi skin lesions that display RB on dermoscopy and their associated characteristics.

Characteristics of the Lesion-Predisposing Factors for RP Appearance	Non-Kaposi Skin Lesion Examples
Nodular subtype—raised lesions	Nodular BCCs, Merkel cell carcinomas, bleeding nodules, pyogenic granola, hypertrophic scars
Lesions with abundant vascularization	-With vascular laminas: vascular lesions such as angiokeratomas, strawberry angiomas, aneurismatic dermatofibroma, pseudo-Kaposi -With network-type abundant vascular pattern: BCCs and scars
Skin lesion compositions	Instances: BCC: basaloid cell islands with abundant vascularization Dermatofibroma: abundance of fibers and collagen
Hematomas	Bleeding nodules or subungual hematoma
Pigment skin—dark-skinned patients	Granulomatous reactions, BCCs, aneurismatic dermatofibroma, pseudo-Kaposi
Depth of the lesion	Deep penetrating nevi, blue nevus
Thickness of lesions	Thick melanomas

## Data Availability

The data described in this study are available upon request from the corresponding author.
